# Image-guided brachytherapy in patients with inoperable FIGO 2009 stage IVB endometrial cancer who were not candidates for palliative surgery

**DOI:** 10.1007/s12094-026-04391-9

**Published:** 2026-06-07

**Authors:** Angeles Rovirosa, Yaowen Zang, Elaine Limkin, Piotr Wojcieszek, Magdalena Stankiewicz, Barbara Segedin, Erik Van Limbergen

**Affiliations:** 1 Fonaments Clinics Department, Faculty of Medicine, Universitat de Barcelona, C/Casanovas 153, 08036 Barcelona, Spain; 2 Radiation Oncology Dpt, Hospital Clinic-Universitat de Barcelona, 08036 Barcelona, Spain; 3 IDIBAPS, 08036 Barcelona, Spain; 4 Cancer Center, Henan Provincial People’s Hospital, Zhengzhou, 450003 China; 5 Radiation Oncology Department, Institut Gustave Roussy, 94805 Villejuif, France; 6 Maria Sklodowska-Curie National Research Institute of Oncology, 44-102 Gliwice, Poland; 7 Radiation Oncology Department, Institute of Oncology Ljubljana, Faculty of Medicine, University of Ljubljana, 1000 Ljubljana, Slovenia; 8 Radiation Oncology Department, University Hospital Gasthuisberg, 3000 Louvain, Belgium

**Keywords:** Image-guided brachytherapy, Figo 2009 stage IVB, Endometrial cancer

## Abstract

**Purpose:**

In patients with endometrial cancer (EC) with metastases at diagnosis, palliative treatment is the only therapy considered and to our knowledge there is no literature on the evolution of these patients following external-beam-irradiation (EBRT) and image-guided brachytherapy (IGBT). We present the clinical outcomes of 11 FIGO 2009 stage-IVB EC patients treated with IGBT ± EBRT.

**Methods/patients:**

From July 2009 to February 2018, 11 stage IVB patients were treated with IGBT ± EBRT in 4 European centres. The treatments and the outcomes of these patients were analysed. Statistics: Kaplan-Meier and descriptive analysis were used for analysing overall survival (OS).

**Results:**

The median age was 63 years (46–79) and the median follow-up was 39 months (8–128). The sites of metastases were reported in 8/11 patients: the lung in 4 patients, peritoneal and liver metastasis in one, pleural metastasis in one, bone in one and inguinal lymph nodes in one. Seven patients underwent chemotherapy (5 achieving complete response (CR)). Two patients received IGBT alone and 9 EBRT + IGBT. Three developed uterine relapse, 3 lymph node relapse and all died. Four had distant metastases and only one was alive after treatment. The OS was 69.3% at 2 and 3 years and 41.6% at 5 and 10 years.

**Conclusion:**

In this series, patients with stage IVB EC with CR after chemotherapy and treated with IGBT ± EBRT achieved an OS of 41.6% at 5 and 10 years. Local treatment with EBRT ± IGBT should be considered mainly in good responders to chemotherapy and should be validated in prospective trials including biological markers.

## Introduction

Endometrial cancer is the most common gynaecological malignancy, the incidence of which is on the rise, particularly among elderly patients [1]. The diagnosis is often made in the early stages, with vaginal bleeding being a common symptom, leading to relatively high survival rates, especially compared to ovarian cancer [2].

Treatment of endometrial cancer typically involves surgery, with or without adjuvant therapies such as vaginal-cuff brachytherapy (VCB) alone, external beam radiation therapy (EBRT) ± VCB, or chemotherapy ± radiotherapy ± VCB, depending on the stage, molecular profile, and estimated risk of recurrence and other prognostic factors such as age [3–5].

With regard to the stages of endometrial cancer, FIGO 2009 stage IVB is the least common (3–13%), mainly in developing countries; however, a recent report by the National Cancer Institute described a rise in the diagnosis of distant metastasis during the COVID-19 pandemic: increasing from 5.2% to 6.5% among females aged 20–49 years during the period from 2019 to 2020, from 7.5% to 8.3% among females aged 50–69 years, and from 10.3% to 11.0% among females aged 70 years and older [1]. In patients with inoperable endometrial cancer, there is a lack of an established treatment protocol. Various therapeutic strategies are used depending on the patient and centre, with chemotherapy and surgery being the preferred methods when feasible. Radiotherapy or other treatments may be considered based on institutional practices and patient response [6]. According to FIGO 2009 staging, the five-year overall survival rates for stage IVB patients receiving these treatments range from 10 to 20% [7, 8].

Anti-PDL-1 agents, such as pembrolizumab, have demonstrated prolonged responses in some metastatic cases. In patients in whom surgery is not feasible, radiotherapy is often recommended as a palliative option following chemotherapy. Nevertheless, there is limited research on the role of radiotherapy in this context, and the lack of definitive evidence prevents consensus on its use [5, 6].

In 2018, the Gynae Working Group of the Group Européen de Curietherapie of the European Society for Radiation Oncology (GEC-ESTRO) conducted a retrospective analysis of medically inoperable stage I–III endometrial cancer patients treated with 3D image-guided brachytherapy (IGBT) ± EBRT. This study included 225 patients, 66 of whom had FIGO 2009 stage I and were treated with exclusive brachytherapy (BT), while 103 patients with stage I–III received EBRT + IGBT. Five-year cancer-specific survival rates were 91% for stage IA, 82% for stage I–II with poor prognostic factors, and 56% for stage III patients treated with EBRT. These findings have led to the inclusion of such treatments in the European Society of Gynaecological Oncology (ESGO)-ESTRO- European Society of Pathology (ESP) guidelines [9]. These results and those from other series were obtained using IGBT ± EBRT, which is at present the elective treatment for inoperable endometrial cancer [10–13].

The current study analyses the outcomes of 11 patients with distant metastases treated with IGBT ± EBRT and systemic therapies, with stereotactic body radiotherapy (SBRT) or conventional radiotherapy to the metastatic sites. Six of these patients have died of cancer, while 3 of the remaining 5 are alive after 7 to 10 years. Here, we present and discuss the results of local radiation treatment in these patients and its role in possibly improving survival.

## Material and methods

The study received Institutional Review Board approval, and all patients provided signed informed consent (HCB/2018/0341).

From July 2009 to February 2018, 11 patients with metastatic endometrial carcinoma (FIGO 2009 stage IVB) considered for palliative treatment were recruited from four European centres. These patients were deemed unsuitable as candidates for surgery by specialised gynaecological cancer tumour boards and, regardless of induction chemotherapy, were treated with IGBT ± EBRT.

The data collected included patient age, FIGO 2009 stage [7], tumour grade, pathological subtype, the largest tumour diameter (mm), diagnostic imaging method, EBRT data, 3D imaging prior to BT, applicator type, imaging used for BT planning, tumour size, gross tumour volume (GTV), and clinical target volume (CTV). Additionally, high-dose-rate vs. pulsed-dose-rate treatment, BT schedule, total BT ± EBRT dose in equivalent 2 Gy fractions (EQD2) using an α/β = 4.5 Gy for CTV and GTV, and α/β = 3 Gy for organs at risk (OARs) were considered. The complications of the treatment were assessed using CTCAE v4 scores [14].

## Statistical analysis

Descriptive analysis was performed and expressed as means, medians and ranges. The Kaplan-Meier method was used to calculate overall survival [15, 16].

## Results

### Patient characteristics

This retrospective descriptive analysis included 11 patients from four European centres: Institut Gustave Roussy in Villejuif (France) – 4 patients, Maria Sklodowska-Curie National Research Institute of Oncology in Gliwice (Poland) – 3 patients, Hospital Clinic-Universitat de Barcelona (Spain) – 3 patients, and Institute of Oncology of Ljubljana (Slovenia) – 1 patient. All patients had distant metastases at the time of diagnosis, with the sites of metastases reported in 8 of the 11 patients: four presented multiple bilateral lung metastases, one had peritoneal and liver metastases, one had pleural metastases, one presented bone metastasis and one had metastasis to the inguinal lymph nodes. All the patients were considered for palliative treatment and received IGBT ± EBRT with curative doses.

The median age of the patients was 63 years (range: 46–79 years); the mean follow-up was 49.4 months and the median follow-up was 39 months (range: 8–128 months). The pathological types were endometrioid in six out of the 11 patients, mixed in 2/11, carcinosarcoma in 1/11 and the remainder presented other pathological types (i.e., neuroendocrine). In terms of locoregional disease involvement, five patients presented different vaginal extension, five in the pelvic nodes, and three with paraaortic node involvement. Table 1 summarises the patient characteristics.

**Table 1:** Patient characteristics

Patient	Age (years)	Sites of metastasis at diagnosis	Histology	Grade/MI	Tumour size (cm)	Extrauterine extension	Lymph nodes and site	Previous chemotherapy treatment and response
1	57	Bilateral lung	Endometrioid	2/≥ 50%	–	Cervix + all vagina	No	Carbo-taxol × 6 PR
2	61	Bilateral lung	Endometrioid	1/≥ 50%	6	Cervix + all vagina	No	Carbo-taxol × 6 CR
3	60	Omentum and liver	Endometrioid	2/< 50%	–	Cervix + 1/3 inner vagina	No	Carbo-taxol × 6
4	69	Pleura	Other	3/–	–	–	No	No
5	80	Not reported	Endometrioid	1/≥ 50%	5	–	Pelvic	No
6	55	Not reported	Endometrioid	2/< 50%	8,3	–	Pelvic + PA	Chemotherapy type not known. CR
7	73	Not reported	Neuroendocrine	3/≥ 50%	6	Cervix + 1/3 vagina + parametrium	Pelvic	No
8	47	Bone	Endometrioid	2/–	–	Cervix + 1/3 vagina	Pelvic + PA	No
9	62	Bilateral lung	Other	–/≥ 50%	3,2	No	No	Carbo-taxol × 6 CR
10	76	Inguinal node	Mixed	3/< 50%	–	No	Pelvic	Carbo-taxol × 6 CR
11	63	Bilateral lung, Omentum	Carcinosarcoma	3/≥ 50%	–	No	Pelvic + PA	Carbo-taxol × 6 PR

*PA* para-aortic, *MI *myometrial invasion, *CR* complete response, *PR* partial response

### Treatment

Before radiotherapy, seven patients underwent 4–6 cycles of chemotherapy, six with carboplatin and paclitaxel and in the other patient the chemotherapy received was not specified. Five of these seven patients achieved complete response and two partial response. Nine patients received EBRT to the pelvic lymph nodes and in five patients the paraaortic field was included, with nodal boost for node-positive patients. An IGBT boost was administered after EBRT while two patients received exclusive BT. Table 2 summarises the treatment administered. EBRT and BT to the CTV doses were almost within guideline recommendations [10, 11, 17]. GTV doses (D90) were reported in 4 cases, ranging from 77.4 Gy to 96.3 Gy EDQ2 (α/β = 4.5). D90 to the CTV was reported in 6 patients, with a median dose of 67.3 Gy (range between 56.4 Gy and 78.4 Gy).

**Table 2:** Treatment characteristics

Patient	EBRT (Gy) EQD2(α/β=4.5)	D90 BT dose EQD2(α/β=4.5) and type of treatment	Overall D90 in CTV EQD2 (α/β=4.5) Gy	Overall D90 in GTV EQD2 (α/β=4.5) Gy	CTV	Technique
1	45 pelvis + 20 boost uterus	NA, HDR	–	–	Uterus, cervix and all vagina	IUT+Vaginal cylinder
2	48.8 pelvis and PA weekly cisplatin	28.6 Gy, HDR	78.4	77.41	Uterus, cervix and all vagina	IUT+Vaginal cylinder+ free hand needles
3	43.6 pelvis	NA, HDR	–	–	Uterus,cervix and 1/3 vagina	IUT+Vaginal cylinder
4	No	NA, HDR	–	96.3	–	Norman Simon
5	43.6 pelvis	24.7 Gy, PDR	71.0	78.7	–	IUT+vaginal mould
6	43.6 pelvis+ Pelvic nodal boost to 56	12.8 Gy PDR	56.4	77.4	–	IUT+vaginal mould
7	43.6 pelvis	14,7 Gy, PDR	58.3	–	Uterus, cervix, parametria and 1/3 vagina	IUT+vaginal mould
8	43.6 pelvis + PA Pelvic and LA nodal boost	30 Gy, PDR	73.6	–	Uterus, cervix and 1/3 vagina	Y-shaped Rotte
9	No	NA	–	–	Uterus	IUT
10	52.6 pelvis + PA Pelvic node boost of 6 Gy	NA	–	–	Uterus	IUT
11	44.6 pelvis+29.6	NA	–	–	Uterus	Y-shaped rote

*PA* paraaortic, *IUT* intrauterine tandem, *NA* not available, *HDR* high-dose rate, *PDR* pulsed-dose rate, *PA* para-aortic, *CTV* clinical target volume, *GTV* gross tumoral volume, *BT* brachytherapy

### Treatment response

The response to the various treatments is shown in Table 3. Five out of seven patients who received systemic treatment achieved complete response after chemotherapy. With a follow-up of 10 and 43 months, respectively, two of the nine patients receiving EBRT developed lymph node relapse in a previously irradiated area (22%), in contrast to neither of the two patients receiving exclusive BT patients. Only four of the 11 patients (27%) developed distant metastases (maxilla, skull bone; nodal relapse in the pelvis, paraaortic and mediastinal areas) during follow-up, and they were treated with various approaches, including intensity-modulated radiotherapy, volumetric modulated arc therapy, SBRT, chemotherapy, and immunotherapy. Two patients (18%) developed uterine recurrence followed by relapse of the previous inguinal node in one and omentum and liver in the other. The EBRT + BT EDQ2_(α/β=4.5)_ D90 to the CTV in these patients was 56.4 Gy and 78.4 Gy.

**Table 3:** Metastases, survival and status

Patient	Uterine relapse and time after treatment	Nodal relapse and time	New distant metastases	Treatment	Follow-up since the first treatment (months)	Status	Cause of death
1	Yes, at 14 months	pelvic at 14 months	No	–	15	Dead	Cancer
2	No	No	Maxilla at 2 m	IMRT with CR (70 Gy)	112	Alive	–
			Skull bone at 8 m	VMAT With CR (30 Gy)			
			Lung at 18 m	SBRT With CR 5 × 5 Gy + Pembrolizumab (PDL-1 +) CR			
3	Yes, 15.07.2020	15.07.2020	Pelvic, PA and mediastinal nodes	Carboplatin + paclitaxel No response	39	Dead	Cancer
4	No	No	No	–	23	Dead	Cancer
5	No	No	No	–	16	Alive	–
6	No	No	No	–	128	Alive	–
7	No	No	No	–	8	Dead	Cancer
8	No	No	No	–	31	Alive	–
9	No	No	No	–	90	Alive	–
10	No	No	Right inguinal node	–	43	Dead	Cancer
11	Hysterectomy at 5 months	PA at 14 months	Liver Omentum	SBRT 24 Gy (3fr) Paclitaxel	42	Dead	Cancer

*IMRT* intensity modulated radiotherapy, *VMAT* volumetric adaptative arc therapy,* SBRT* stereotactic body radiotherapy, *CR* complete response, *PA* paraaortic

### Survival

**Fig. 1:**
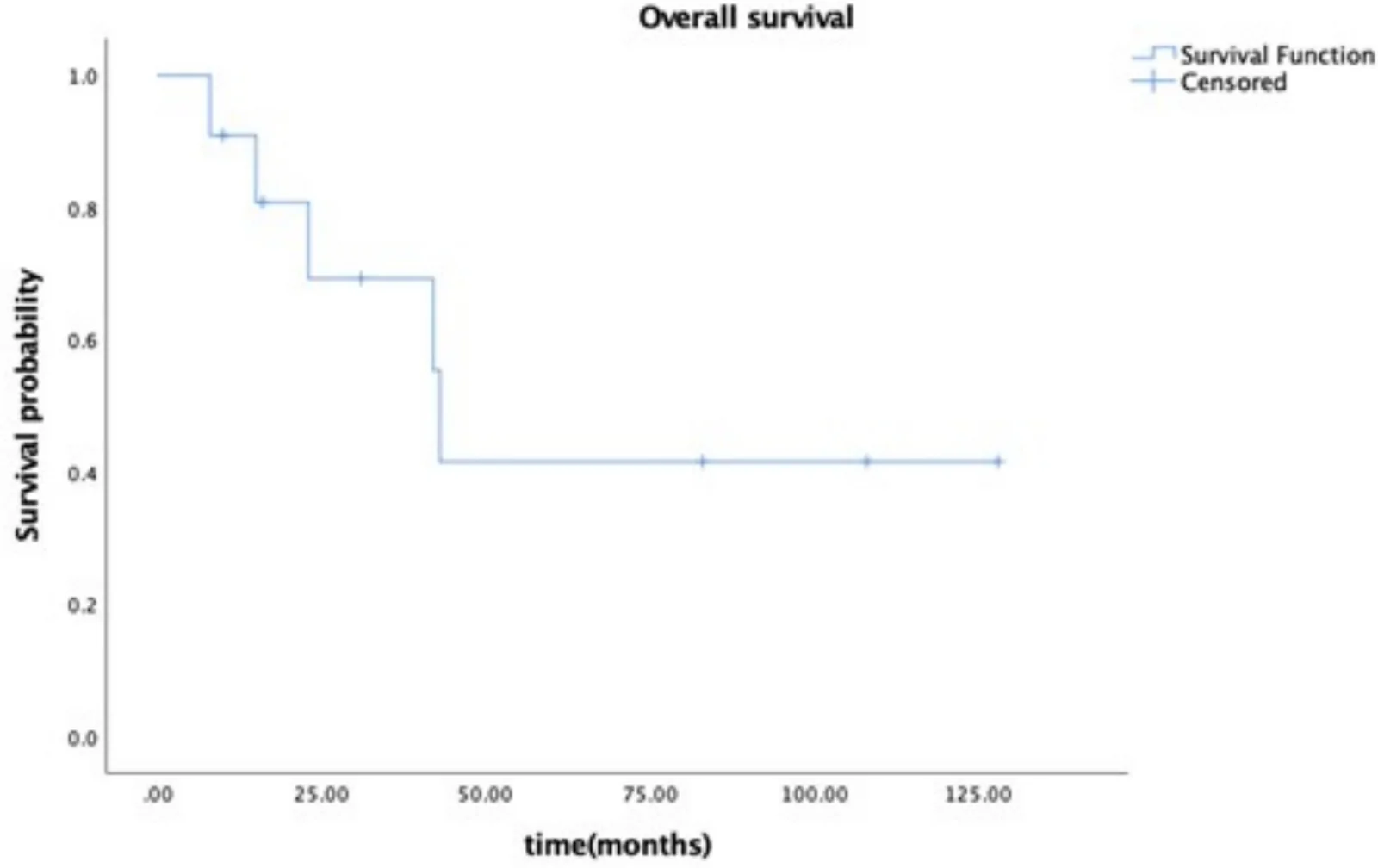
Overall survival of the patients at 10 years

The mean survival for the entire cohort was 49.8 months, and the median survival was 39 months (range: 8–128 months). The mean survival for the six patients who died from cancer was 28.3 months, with a median survival of 31 months (range: 8–43 months). The five patients (55.5%) who remained alive without evidence of cancer presented a mean survival of 75.4 months and a median survival of 90 months (range: 16–128 months). Figure 1 illustrates the overall survival rates, which were 69.3% at 2 and 3 years and 41.6% at 5 and 10 years. Therefore, both the mean and median survival were significantly higher in patients without recurrence compared to those presenting relapse and subsequently dying. With the present follow-up, no patient died from causes other than cancer.

### Late secondary effects

Treatment-related late secondary effects were reported in 10 out of the 11 cases. Rectal complications included one Grade 3 (10%) and one Grade 1 toxicity (10%), while sigmoid complications included one Grade 2 (10%) and one Grade 1 (10%).One patient presented Grade 2 small bowel toxicity (10%) and 2 patients had vaginal complications that included one Grade 2 (10%) and one Grade 4 toxicity (10%). No bladder complications were reported (Table 4).

**Table 4:** Characteristics of the patients alive after treatment

Patient	Uterine relapse and time after treatment.	Nodal relapse and time	Response to Chemotherapy/radiotherapy of distant metastases	New distant metastases	Treatment	Follow-up since the first treatment (months)	Status
2	No	No	Yes, CR	Maxillary, skull 2 sites lung M1	IMRT with CR IMRT With CR SBRT With CR + Pembrolizumab (PDL-1)	112	Alive
5	No	No	No chemo bone	No	–	16	Alive
6	No	No	CR	No	–	128	Alive
8	No	No	CR	No	–	31	Alive
9	No	No	CR	No	–	90	Alive

*CR* complete response, *IMRT* intensity modulated radiotherapy, *SBRT* stereotactic body radiotherapy

## Discussion

The increase in life expectancy in developed countries has contributed to an increase in the number of cases of endometrial cancer, particularly among older patients. Due to comorbidities, some of these patients are not deemed suitable candidates for surgery. Endometrial cancer with distant metastasis at diagnosis is typically treated palliatively with chemotherapy ± immunotherapy. However, patients achieving complete response to systemic treatment may be candidates for surgery or other local therapies, including EBRT and/or BT. The use of immunotherapy and new therapeutic targets has expanded the options for local therapies followed by systemic treatment.

Very few studies have analysed inoperable metastatic endometrial cancer treated with 3D IGBT, and it remains a relatively underexplored area. A systematic review by Capozzi et al. [5] published in 2023 included patients treated with surgery ± other adjuvant therapies. The overall survival was 34.3 months for optimally debulked patients and only 11 months for those with residual disease > 1 cm. Other studies confirmed these findings but with lower survival rates (14.5 to 25 months) [18–22]. In the study by Capozzi et al*.,* age over 60 years and serous pathology was associated with a poorer prognosis. Additionally, patients with distant metastasis exhibited a better prognosis compared to those with peritoneal metastasis, particularly in cases in which cytoreduction was not feasible [5].

In the current series, 11 patients with stage IVB (FIGO 2009) metastatic endometrial cancer were treated with IGBT ± EBRT, which was performed after chemotherapy in 7 patients. Two of four patients who did not receive chemotherapy died, and 2 remained alive at 16 and 31 months of follow-up. In the patients receiving preoperative chemotherapy, the survival at 2 and 3 years was 69% and 41.6%, respectively. The survival of the patients remaining alive at 5 and 10 years suggests that personalised approaches could improve outcomes. The median survival of the patients who died from cancer was 31 months compared to 90 months among those remaining alive. The best outcomes were observed in patients presenting complete response to chemotherapy, low grade disease, complete uterine response, and in patients successfully treated for new metastases with radiotherapy or pembrolizumab, with four patients surviving a median of 90 months. For patients lacking these factors, a 31 month survival is significant compared to untreated individuals, with anticipated improvements in quality of life [6].

The present study has some limitations, such as its small retrospective design, the lack of some information in certain patients and the heterogeneity among the treatments. However, this is the first in the literature to present curative treatment options for patients with metastasis at diagnosis, who were subsequently treated using 3D-IGBT ± EBRT. Not all patients may be candidates for this approach, but this treatment option may be considered in cases with good response to systemic treatment, low disease burden, and good performance status. In this study, the patients were highly selected, which may explain the good results despite some heterogeneity in treatments. Another limitation of this exploratory study was the small number of patients included which limited the statistical analysis and producing a potential selection bias with and overinterpretation of the results. Considering the number of patients in this series, we cannot conclude that local treatment (including IGBT??) is beneficial in metastatic patients, but rather that some selected patients may benefit from it, highlighting the role of local control of symptoms. Further prospective studies are necessary to identify which patients with distant metastasis at diagnosis are eligible for a more aggressive treatment approach.

This series is the first to report that inoperable stage IVB patients can be treated with IGBT ± EBRT, leading to either curative outcomes or prolonged survival as palliative treatment.

## Conclusion

Treatment with IGBT ± EBRT for stage IVB inoperable endometrial cancer showed promising results, with an overall survival rate of 41.6% at 5 and 10 years, respectively in patients achieving complete response to chemotherapy before local treatment.

This study shows that survival was high in patients without progression after treatment, while reasonable disease control was achieved in the remaining patients. The overall survival might have been higher if only the endometroid type of cancer had been considered.

Future advances in systemic treatments and the analysis of biological markers may likely improve survival outcomes in these patients.

## Data Availability

Not applicable.
